# Is Acupuncture Safe and Effective Treatment for Migraine? A Systematic Review of Randomized Controlled Trials

**DOI:** 10.7759/cureus.20888

**Published:** 2022-01-03

**Authors:** Noreen Naguit, Sadia Laeeq, Rakesh Jakkoju, Tiba Reghefaoui, Hafsa Zahoor, Ji Hyun Yook, Muneeba Rizwan, Noor ul ain Shahid, Lubna Mohammed

**Affiliations:** 1 Department of Research, California Institute of Behavioral Neurosciences & Psychology, Fairfield, USA

**Keywords:** acupoints, needle therapy, migraine without aura, migraine with aura, acupuncture, episodic migraine, chronic migraine, migraine disorder

## Abstract

Migraine is a debilitating condition that places a substantial economic burden on society and seriously affects patients' quality of life. Currently, there is no known "cure" for migraines, and pharmacologic treatments or prophylaxis carry many unwanted effects. Acupuncture has been accepted as an alternative treatment. However, its effectiveness is still debated. This is a systematic review of randomized controlled trials (RCT) that investigate acupuncture safety and efficacy in migraine versus various control groups. We searched PubMed, Google Scholar, Science Direct, and Cochrane library using keywords: migraines, migraine with aura, migraine without aura, headache, acupuncture, and needling therapy. Two independent reviewers participated in data extraction and assessment. Fifteen randomized controlled trials involving 2,056 participants that met the inclusion criteria were obtained and analyzed. Based on the findings, seven out of 10 trials that compared acupuncture with sham acupuncture showed a more significant reduction in the frequency of migraine attacks and headache intensity. Four studies revealed acupuncture is just as effective and has fewer side effects than any western medicine. Acupuncture can be recommended as an alternative or adjunct to drug treatment for patients suffering migraines. However, further clinical trials that utilized the Standards for Reporting Interventions in Clinical Trials of Acupuncture (STRICTA) recommendation are still needed to strongly present an evidence-based strategy.

## Introduction and background

Migraine is a common neurological disorder characterized by severe, recurrent, unilateral, and throbbing headaches, often accompanied by neurological and systemic symptoms [1]. It is a debilitating condition that places a substantial economic burden on society and seriously affects patients' quality of life [2,3]. The Global Disease Burden (GBD) 2019 study reported that migraine remains second among the world's causes of disability and first among young women [4]. The GBD survey in 2017 revealed that 1.25 billion people had a migraine [5]. According to the World Health Organization (WHO), it is one of the most serious, long-lasting, and disabling disorders with prevalence equal to quadriplegia, mental illnesses, and dementia [6].

Migraine has two major types: migraine without aura (MWOA) and migraine with aura (MWA). Migraine without aura is a clinical syndrome characterized by recurrent headaches with attacks lasting 4-72 hours, often associated with nausea, photophobia, or phonophobia. In comparison, MWA is a recurrent attack lasting minutes and characterized by transient neurological symptoms (visual, sensory, motor, retinal, and speech or language) that usually precede or sometimes accompany the headache. In addition, migraines can be differentiated as chronic and episodic. These are part of the spectrum of disorders, but they are distinct clinical entities. International Headache Society (IHS) currently defines chronic migraine as a headache for at least 15 days per month, with migraine feature on eight of those days. Those with migraines who have 0-14 headache days per month characterize episodic migraines. The accurate diagnosis is based upon the clinical presentation and ruling-out other headache disorders [7,8]. 

Currently, there is no known "cure" for migraines. Medical treatment can be acute (abortive) or preventive; a frequent severe headache may require both approaches. Several drugs can be used to lessen the frequency of acute migraine episodes: aspirin, acetaminophen, ergotamine, or triptans, and non-steroidal anti-inflammatory drugs (NSAIDs) [9,10]. The recommended preventive therapy includes metoprolol, timolol, propranolol, divalproex sodium, sodium valproate, and topiramate [10]. They provide some relief, but they are associated with adverse events such as hypotension, nausea, depression, drowsiness, cardiovascular effects, gastrointestinal intolerance, and rarely kidney damage, leading to patients' poor compliance [1,6,9]. In addition, a medication-induced migraine may result from unwarranted use of these painkillers or specific anti-migraine [9]. Therefore, more patients are seeking alternative treatment or non-drug interventions [11]. 

In the last decade, a known non-pharmacological treatment for migraine prophylaxis is acupuncture. Over 3,000 years, it has been one of the main treatments of traditional Chinese medicine (TCM) [6]. National Institutes of Health in 1998 suggested acupuncture as an alternative treatment for headaches, and it has been utilized as a traditional remedy for migraines in China. In the method thin needles are inserted into acupoints, which are specific points along energy meridians. However, despite the popularity of acupuncture in migraine therapy, there is limited knowledge about its physiological mechanisms. Some research reported that it could cause inhibition in pain transmission within the central nervous system by stimulating different types of afferent nerves. The release of some pain suppressors such as endorphin, serotonin, dopamine, neurotrophins, and nitric oxide in the brain is also facilitated. In addition, this method also decreases serum matrix metalloproteinase that causes relief in migraine headaches [1]. 

Acupuncture has been effectively used in clinical practice to prevent and treat migraines and is becoming accepted in Western countries [11]. The duration and frequency of both chronic and acute migraines have been reported to be reduced in acupuncture. Multiple studies also noted that it was at least non-inferior to standard drug therapy and had some levels of superiority over sham acupuncture [12]. This systematic review aimed to assess the efficacy and safety of acupuncture in the treatment of migraine in comparison with pharmacological or placebo treatment.

## Review

Methods

The primary purpose of this study was to provide a systematic review on the efficacy and safety of acupuncture treatment for migraine compared to various control groups. The review was conducted in adherence with the recommendation of the preferred reporting items for systematic reviews and meta-analyses (PRISMA) guidelines 2020 [13] and updated some search methods with PRISMA-S checklist 2021 to ensure high-quality review.

Search Strategy

We systematically searched PubMed, Google Scholar, Science Direct, and the Cochrane library until August 22, 2020. To precisely collect all potentially relevant articles in assessing acupuncture as an alternative or additional migraine treatment, we use appropriate keywords and medical subject headings (MeSH) terms. The keywords used were: migraines, migraine with aura, migraine without aura, headache, acupuncture, and needling therapy. We used the Boolean scheme to galvanize the keywords and MeSh strategy format and subsequently employed it in PubMed. The search strategy and keywords are mentioned in Table 1. All articles were later retrieved, and references were thoroughly checked to avoid neglecting any potential relevant articles; thereafter, the title, abstract, and subject headings were screened for relevance. 

**Table 1:** MeSH strategy combined with keywords

MeSH and Keywords search	Database	Number of Results	Inclusion/ Exclusion	Duplicates removed
Migraine OR Migraine without aura OR Migraine with aura OR Headaches ("Migraine Disorders"[Majr]) OR ( "Migraine Disorders/analysis"[Majr] OR "Migraine Disorders/cerebrospinal fluid"[Majr] OR "Migraine Disorders/chemically induced"[Majr] OR "Migraine Disorders/complications"[Majr] OR "Migraine Disorders/diagnosis"[Majr] OR "Migraine Disorders/drug therapy"[Majr] OR "Migraine Disorders/epidemiology"[Majr] OR "Migraine Disorders/genetics"[Majr] OR "Migraine Disorders/immunology"[Majr] OR "Migraine Disorders/metabolism"[Majr] OR "Migraine Disorders/pathology"[Majr] OR "Migraine Disorders/physiology"[Majr] OR "Migraine Disorders/physiopathology"[Majr] OR "Migraine Disorders/prevention and control"[Majr] OR "Migraine Disorders/rehabilitation"[Majr] OR "Migraine Disorders/statistics and numerical data"[Majr] OR "Migraine Disorders/urine"[Majr] ) AND Acupuncture analgesia OR Acupuncture OR needling therapy ( "Acupuncture Therapy/adverse effects"[Majr] OR "Acupuncture Therapy/classification"[Majr] OR "Acupuncture Therapy/education"[Majr] OR "Acupuncture Therapy/epidemiology"[Majr] OR "Acupuncture Therapy/history"[Majr] OR "Acupuncture Therapy/instrumentation"[Majr] OR "Acupuncture Therapy/methods"[Majr] OR "Acupuncture Therapy/mortality"[Majr] OR "Acupuncture Therapy/nursing"[Majr] OR "Acupuncture Therapy/pharmacology"[Majr] OR "Acupuncture Therapy/standards"[Majr] OR "Acupuncture Therapy/statistics and numerical data"[Majr] OR "Acupuncture Therapy/therapeutic use"[Majr] OR "Acupuncture Therapy/therapy"[Majr] OR "Acupuncture Therapy/trends"[Majr] )	PubMed	7,752	377	10

Criteria for considering studies

Type of Studies

We only included published randomized controlled trials (RCT) between 2011 and 2021 in the English language and humans.

Types of Participants

Participants are over the age of 18 who had been clinically diagnosed with migraine (criteria were not limited), including migraine with or without aura; episodic migraine; chronic migraine; and acute migraine. 

Studies that used acupuncture on tension headache, cluster headache, and menstrual migraine were excluded.

Types of Intervention

There was no restriction on the types of acupuncture treatment: manual acupuncture (MA), electroacupuncture (EA), auricular acupuncture (AA), while the control groups were treated with pharmacologic medication, sham acupuncture/placebo acupuncture (needle is penetrated in the non-acupuncture point), or no treatment. 

Types of Outcome Measures

The primary outcome was pain score after the intervention as measured by visual analogue scale (VAS) or other validated scales. Pain relief as documented on the patient's headache diary (pain intensity, attack frequency, duration). The secondary outcome was the use of rescue medication and the quality of life measured by validated scales. 

Data Extraction

Two researchers (NN, SL) autonomously carried out the data selection and extraction. If disagreement occurred at any stage, a third author considered the available information, or if necessary, the study authors were contacted for clarification. When eligibility could not be determined in cases of disagreements, both researchers discussed the study based on the relevance to inclusion and exclusion criteria, intervention used, and outcome measured to reach an accord. We obtained the aid of a third reviewer in instances when common ground could not be attained.

Study Quality Appraisal

The clinical trials were critically evaluated with the Cochrane risk bias tool [14]. Each study was scrutinized based on seven domains: random sequence generation, allocation concealment, blinding of participants and personnel, blinding of outcome assessment, incomplete outcome data, and other sources of bias. The risk of bias in each domain was assessed as high, low, unclear, or no information. A study was judged as having an overall low risk of bias if each domain was gauged to have a low risk of bias. Otherwise, it was considered as having an overall high risk of bias. 

**Figure 1:**
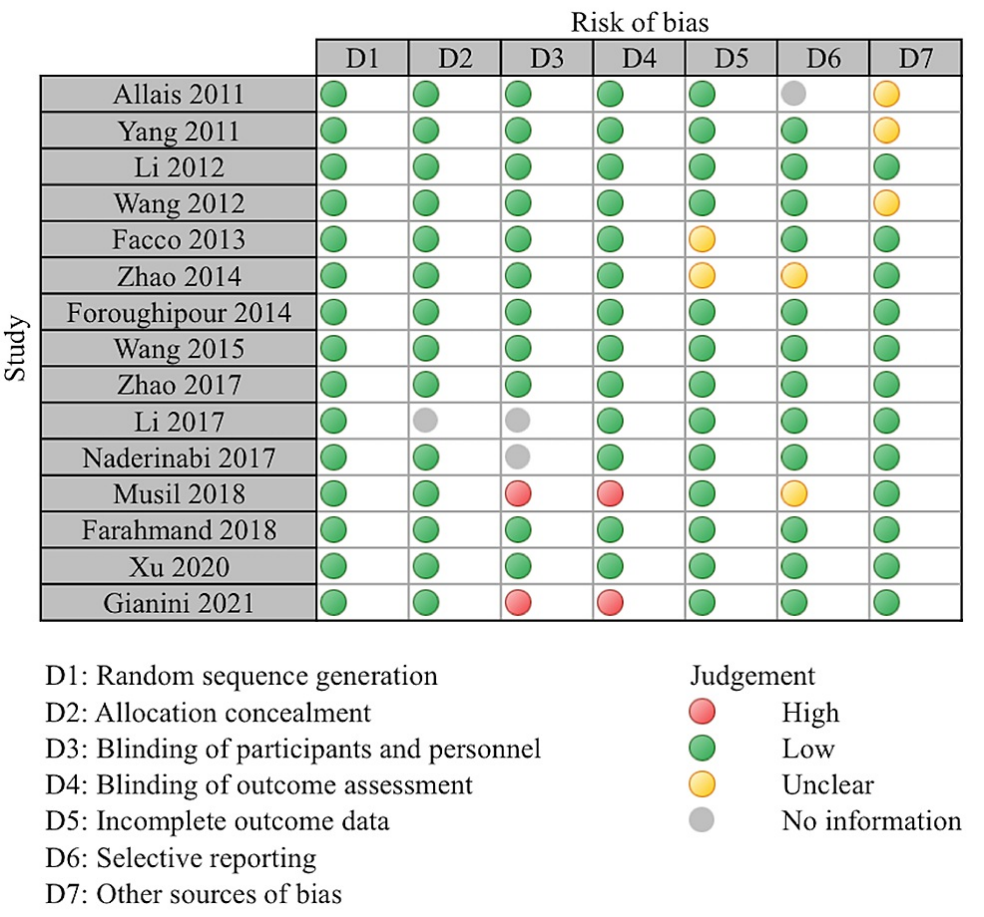
Risk of bias assessment

All the included studies mentioned randomization. Fourteen of the studies reported appropriate allocation concealment. Most of the studies were single-blinded on the part of the participants. While blinding both participants and personnel is challenging due to the nature of acupuncture intervention. However, 11 trials were assessed as low risk as this study mentioned efforts to minimize performance bias. Two studies were open-label trials as the patient knew the intervention being done and were both judged as high risk. Thirteen studies mentioned the study outcomes and were assessed as low risk, and two were assessed as an unclear bias. Thirteen studies were assessed as low risk on selective reporting, while two were unclear, and one study did not report the information. Other sources of bias were assessed as low risk in 12 studies, while three trials were judged with unclear risk of bias. A summary of the Cochrane risk bias tool is given in Figure 1.

Results

Study Selection

A total of 21,678 articles were found using keywords and MeSH terms. Out of 21,678 articles, 7,752 were from PubMed, 3,523 from Science Direct, 10,400 from Google Scholar, and three from Cochrane Library; 547 articles remained after applying the criteria above. All articles found based on the search results were saved into EndNote (Clarivate Analytics, London, UK). We then filtered the remaining articles based on the title's relevance and contents of their respective abstract to our ongoing research. Out of which, 506 were discarded due to irrelevance, and 10 were duplicated articles.

**Figure 2:**
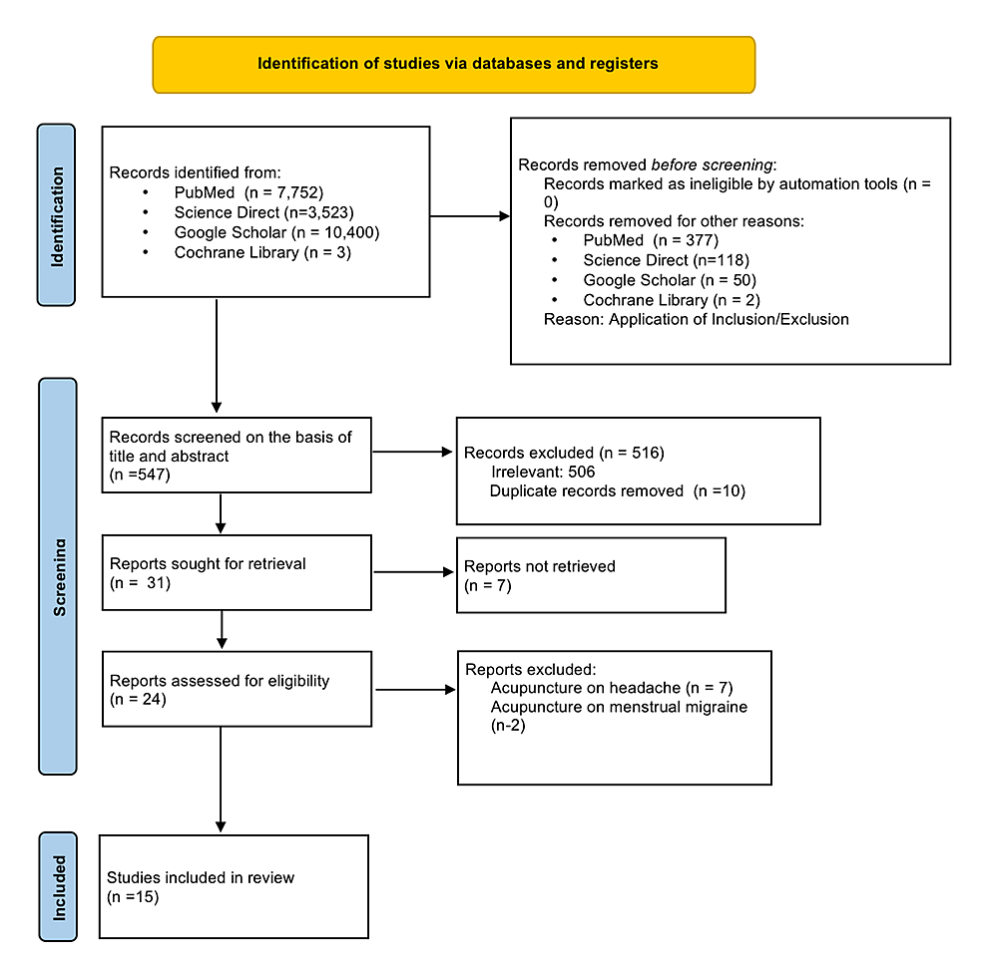
PRISMA flow diagram PRISMA: preferred reporting items for systematic reviews and meta-analyses.

Hence, 31 articles were left, and we checked for availability of full texts, out of which seven articles were removed. In addition, nine articles were rejected as these focused on acupuncture on menstrual migraine, tension, and cluster headache. Fifteen clinical trials were finalized. Complete preferred reporting items for systematic reviews and meta- analyses (PRISMA) flow diagram is shown in Figure 2.

Study Characteristics

We identified 15 eligible trials involving a total of 2,056 participants, which were eligible for inclusion in this review. Treated patients underwent manual acupuncture (MA), electro-acupuncture (EA), auricular acupuncture (AA), and control patients received no treatment/waiting list, sham acupuncture, or pharmacological medications. Of the 15 trials, seven were conducted in China [9,11,15-19]; three in Italy [20-22]; three in Iran [1,2,23]; one in Australia [24], and one in Czech Republic [25]. Seven studies had participants with MWOA only [11,17-21,23]; two with an acute attack [1,9]; two with chronic migraine [2,15]; four with MWA/MWOA [16,22,24,25]. Table 2 summarizes the characteristics of the included studies.

**Table 2:** Summary of the included studies AA = auricular acupuncture, AE = adverse effect, BDI = Beck depression inventory, CM = chronic migraine, CG = control group, EA = electro-acupuncture, HA = headache, HADS = hospital anxiety and depression scale, HC = healthy control, MA = manual acupuncture; MIDAS = migraine disability assessment scale; MWA = migraine with aura; MWOA = migraine without aura; MSQ = migraine-specific quality-of-life questionnaire; NR = not reported, PRS = pain relief score, PSQ1 = Pittsburgh sleep quality index; SA = sham acupuncture; SAS = self-rating anxiety scale; SDS = self- rating depression scale; SPLS = six-point Likert scale; SQ = subcutaneous, TG = treatment group, Tx = treatment, VAS = visual analogue scale, WL = waiting list

Study	Country	Condition	Number of Participants	Age in years (Mean or Median)	Experimental Intervention (n)	Control Intervention(n)	Outcomes Measured	Results	Adverse Effect
Allais et al., 2011 [20]	Italy	MWOA	94	TG: 35.93	AA (46)	Placebo AA (48)	VAS	There was a significant reduction of VAS score value in TG (<0.001) at 10, 30, 60 and 120 min after needle insertion. No significance observed in placebo AA.	NR
				CG: 33.2					
Yang et al., 2011 [15]	China	CM	66	TG: 47.6	MA (33)	Topiramate 25mg/day for one week then weekly increase of 25mg up to 100 mg/day (32)	Mean HA, MIDAS, BDI, HADS	There was a significantly larger decrease in mean monthly number of moderate/severe HA in TG compared to CG (<0.1).	TG: 6% reported AE which is related to local insertion of needles (pain, ecchymosis and local paraesthesia)
				CG: 48.1					
Li et al., 2012 [16]	China	MWA, MWOA	480	36.9	EA (326)	SA (118)	Frequency of Migraine, VAS	Patient on TG reported fewer days with migraine in week 5-8 but not statistically significant (p>0.05). There was a significant reduction in number of days with migraine in TG vs. CG during week 13-16 (p= 0.003)	37 AE (25 SQ hemorrhage, 6 SQ hematoma, SQ 5 ecchymosis and 1 leg weakness)
Wang et al., 2012 [9]	China	Acute attack	150	TG: 37.8	MA (75)	SA (75)	VAS	There was a significant difference in mean VAS score between TG and CG (p= 0.001)	MA: mild AE
				CG: 38.6					SA: 4 mild AE (Little bleeding after removal of needle, fatigue)
Facco et al., 2013 [21]	Italy	MWOA	100	TG: 40	MA (41)	Valproic acid 600 mg/day(41)	MIDAS, PRS	Pain intensity was better in TG at three months (p= 0.0001) but at six months pain intensity and PRS were better in TG.	TG: No AE
				CG: 34					CG: 20 patients reported mild AEs (5 nausea, 5 constipation 5 abdominal pains, 3 drowsiness, 3-weight gain and 1 itching)
Zhao et al., 2014 [17]	China	MWOA	80	TG: 33.35	EA (40)	Placebo EA(40)	VAS	There was a significant difference in VAS score between the two groups (P=0.015). No significant differences were observe between the two groups for the frequency of migraine attack per four weeks at the end of treatment (p=0.05).	TG: 1 fainting during acupuncture treatment, minor hemorrhage at needling site
				CG: 33.23					
Foroughipour et al., 2014 [23]	Iran	MWOA	100	36.5	MA (50)	SA (50)	HA attack per month	There was a significant difference in the frequency of attacks in TG vs. CG (p <0.001) after one and two months of treatment. Frequency increased in months three and four but significantly lower than baseline.	NR
Wang et al., 2015 [24]	Australia	MWA, MWOA	50	TG: 41.6	MA (26)	SA (24)	VAS, SPLS	The TG reported significantly less migraine days (p= 0.0008), less severe migraine (p=0.004), and increased pressure pain thresholds compared to CG.	MA: 37 mild to moderate AEs out of 400 sessions (none required medical intervention)
				CG: 43.8					
Zhao et al., 2017 [18]	China	MWOA	249	38.1	EA (83)	SA (83)	MSQ, SAS,	The mean change in frequency of migraine attacks differs significantly in the three groups at 16 weeks. A greater reduction was observed in TG compared to SA (p=0.002) and in TG vs. WL group (p 0.001). There was no statistical difference between SA and WL group (p=0.07).	Seven patients (five in Acupuncture and two in Sham Acupuncture) reported AEs during 24 weeks
						WL (83)			Tingling sensation on acupoints and swelling of left ankle after needle was removed
Li et al., 2017 [19]	China	MWOA	106	21.29	MA (35)	SA (11)	VAS, SAS, SDS	There was significant improvement in VAS score (<0.05) in TG. CG showed insignificant improvement in VAS score and HA frequency (P>0.05). However, there were insignificant differences in changes in VAS score, changes in HA frequency, SDS, and SDS improvement (<0.05). There was significant therapeutic effect of TG compared to WL group in VAS score and HA frequency (p < 0.05).	NR
						HC (42)			
						WL (16)			
Naderinabi et al., 2017 [2]	Iran	CM	150	TG: 37.2	MA (50)	Botulinum Toxin A (50)	VAS	Pain severity significantly decreased in three groups (p=0.0001), with greater reduction in TG (p =0.0001).	Acupuncture: bleeding/ hematoma
				CG 1: 36.8		Sodium Valproate 500 mg (50)			Botox: ptosis, facial masking or asymmetry
				CG 2: 37.6					Valproate: asthenia, anorexia, weigh gain, tremor, insomnia, somnolence, alopecia
Musil et al., 2018 [25]	Czech	MWA, MWOA	86	TG: 45.6	MA (42)	WL (44)	VAS, MIDAS	There was a reduction in migraine days in TG (5.5 days) and WL (2.2 days), with statistical significant of 2.0 migraine days (95% CI: -4 to -1) after 12 weeks of acupuncture. A significantly greater reduction in number of migraine days per four weeks in TG vs. CG (95% CI: -6 to -2).	Facial hematoma resolved in two days without intervention.
				CG: 46.5					
Farahmand et al., 2018 [1]	Iran	Acute Migraine Attack	60	31.4	AA (30)	Placebo AA (30)	VAS	There was a significant difference between two groups on checkpoint of 15, 30, 45, and 60 minutes after acupuncture (p=0.05); however pain scores were not statistically different between two groups on two, three, and four hours after intervention (>0.05).	NR
Xu et al., 2020 [11]	China	MWOA	150	TG: 36.6	MA (60)	SA (60)	VAS, MSQ PSQ1, MIDAS, BAI, BDI-II	MA resulted in a significant greater reduction in migraine days compared to SA at 13 to 20 weeks and a significantly greater reduction in migraine attacks at weeks 17 to 20.	TG: Five reported at least one acupuncture related AE
				CG 1: 36		Usual Care (30)			
				CG 2: 37.3					
Giannini et al., 2021 [22]	Italy	Episodic Migraine: MWA/MWOA	135	TG: 33.6	MA (69)	Pharmacologic Treatment (66)	HA diary difference in # of days/MIDAS	HA frequency decreased significantly after treatment without difference in the two groups (p=0.556).	NR
				CG: 34.7					

Acupuncture Interventions

**Figure 3:**
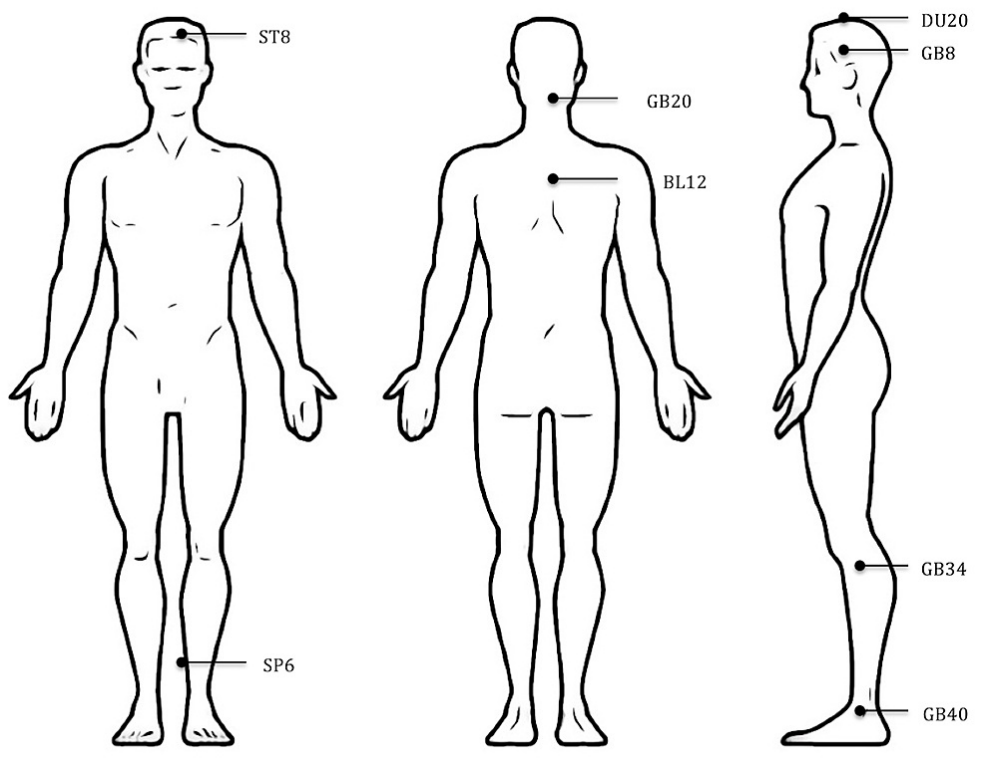
Approximate location of the most frequently used acupoints in the included studies

Of the 15 trials, 10 used MA [2,10,11,15,19, 21-25] three used EA [16-18] and two used AA [1,20]. The number of acupoints used varied from one to 25. Figure 3 shows the approximate location of the most frequently used acupoints in the included studies: GB 20 Fengchi, GB 8 Shuaigu, GB 34 Yanglingchuan, GB 40 Qiuxu, ST8 touwei, SP6 Sanyinjiao, DU 20 Baihui, and BL 12 Fengmen.

Treatment duration ranged from 25 to 30 minutes. De-qi sensation (a sensation of soreness, numbness, distention, or radiating that indicates effective needling) was performed in most trials, but three studies did not mention De-qi sensation [1,20,22]. Table 3 shows the acupuncture intervention of the included studies based on the standards for reporting interventions in clinical trials of acupuncture (STRICTA) guidelines [26].

**Table 3:** Acupuncture interventions of the included studies based on STRICTA recommendations NR = not reported; STRICTA = standards for reporting interventions in clinical trials of acupuncture

Study	Name of Acupoints	Number of Needle	Depth of Insertion	De-qi Response	Number of Treatment Session	Duration and Frequency of Treatment Session	Needle Retention
Allais et al., 2011 [20]	Auricle (M), Sciatic nerve (S)	4	NR	NR	NR	NR	Semi-permanent
Yang et al., 2011 [15]	Bilateral Cuanzhu, Fengchi, Taiyang, Yintang	7	Standard to each point accdg, to classic acupuncture point.	De-qi	24	30 minutes each, twice a week	30 minutes
Li et al., 2012 [16]	Waiguan (TE5), Yanglingquan (GB34), Qiuxu (GB40), Fengchi (GB20)	4	NR	De-qi	20	30 minutes/one per day for five consecutive days	NR
Wang et al., 2012 [9]	DU20, DU24, ST8, GB8, GB20, SJ5, GB34, LI4, ST44, BL60, SI3, LR3, GB40, PC6	10-12	10-15 mm	De-qi	1	30 minutes	NR
Facco et al., 2013 [21]	GB20, St8, EX-HN5, GB8, BL12, BL60, TE5, GV14, St40, SP6, CV12, LR3, LR4, St40, SP9,GV23, CV12, GB12	NR	Classical prescription of acupoints	De-qi	20	30 minutes each, twice per week	30 minutes
Zhao et al., 2014 [17]	SJ5 (Waiguan), GB20 (Fengchi), GB34 (Yanglingquan), GB40 (Qiuxu); SJ22 (Erheliao), PC7 (Daling), GB37 (Guangming), SP3 (Taibai)	8	25-35 mm	De-qi	32	30 minutes/4x a week	NR
Foroughipour et al., 2014 [23]	Shaoyang, Yangming, Taiyang, Jueyin	NR	NR	De-qi	12	30 minutes 3x/week	NR
Wang et al., 2015 [24]	Fengchi (GB20, bilateral), Taiyang (EX-HN5), Shuai Gu (GB8), Hegu (LI4), Baihui (DU20), Xingjian (LR2), Taichong (LR3), Taixi (KI3), Xuanzhong (GB39), Sanyinjiao (SP6)Baihui (DU20), Shang Xing (DU23), Zusanli (ST36), Sanyinjiao (SP6), Feng Long (ST40), Zhongwan (CV12), Yinlingquan (SP9)Sanyinjiao (SP6), Xuehai (SP10), Ashi point	9-12	10-30mm	De-qi	16	2x/week for four weeks. Once every two weeks for four weeks and once a month for two months	25 minutes
Zhao et al., 2017 [18]	GB20, GB8, SJ5, GB34, BL60, SI3, LI4, ST44, LR3, GB40	4	NR	De-qi	20	30 minutes each/ 5 x a week	NR
Li et al., 2017 [19]	Yanglingquan (GB34), Qiuxu (GB40), Waiguan (SJ5), Xiyangguan (GB33), Diwuhui (GB42), Sanyan- gluo (SJ8), Zusanli (ST36), Chongyang (ST42), Pianli (L16), NAP1, NAP2, NAP3.	6	5-15 mm	De-qi	20	30 minutes/5x per week	NR
Naderinabi et al., 2017 [2]	Gallbladder (GB) 41, GB 20, GB 15, GB14, GB10, GB8, large intestine (LI) 4, liver 3, Sanjiao 5, Du-Mai 20, 2 Taiyang	10-12	10-15 mm	De-qi	30	Once every two days	NR
Musil et al., 2018 [25]	Fengchi (gB20) Taiyang (eX-hN5) shuai gu (gB8); hegu (li4); Baihui (DU20), Xingjian (lr2), Taichong (lr3), Taixi (Ki3), Xuanzhong (gB39), sanyinjiao (sP6); Baihui (DU20), shang Xing (DU23), Zusanli (sT36), and sanyinjiao (sP6); Feng long (sT40), Zhongwan (cV12), and Yinlingquan (sP9); sanyinjiao (sP6), Xuehai (sP10), ashi point	9-12	10-30 mm	De-qi	14	25 minutes each, 2x/week or once a week during week 5-8 and once every 14 days during the last month	25 minutes
Farahmand et al., 2018 [1]	Ear acupoints (shen men, autonomic, thalamus, frontal, and temple)	NR	NR	NR	NR	NR	NR
Xu et al., 2020 [11]	L14, LR3, EX-HN5, GB20, GB8, ST8, BL10, DU20	NR	NR	De-qi	20	30 minutes	10 seconds and repeated 4x with intervals of 10 minutes
Giannini et al., 2021 [22]	LR 3 (taichong), GB 34 (yanglingchuan), SP 6 (sanyinjiao), LI 4 (hegu), TE 5 (weiguan), GV 20, ST 8 (touwei), BL 2 (zanzhu), GB 4 (hanyan), GB 8 (shuaigu), GB 20 (fengchi), BL 12 (fengmen)	NR	NR	NR	12	NR	NR

Of the 10 trials that used MA, one compared MA to no treatment or WL [25], five studies compared MA to sham acupuncture (needles do not go as deep or inserted in non-acupoint) [9,16,19,23,24], and four studies compared MA to pharmacological medications [2,15,21,22]. Of the three trials that used EA, one compared EA to SA [16], another study compared EA to placebo EA [17] and one study compared EA to SA and no treatment/WL [18]. Two trials that used AA used placebo AA as a control group [1,20]. All of the placebo controls used non-acupoint acupuncture.

Discussion

Acupuncture has been a widely accepted alternative treatment for chronic pain, and it involves inserting needles into the specific acupoints on the patient's body. During the last decade, there was a substantial development on how acupuncture provides analgesia. Analgesia is promoted when soreness, numbness, heaviness, and distension are noted following acupuncture manipulation [27]. A significant way to objectively assess the therapeutic effect of acupuncture and make it internationally accepted is by developing high-quality clinical research on acupuncture. It is also an important way to support the development of acupuncture treatment standards [28]. This systematic review of randomized controlled trials was aimed to summarize and evaluate the effectiveness and safety of acupuncture treatment on migraines.

Analgesia in Acupuncture

In this review, most studies used MA as an intervention, and its analgesic effect could be explained by C-fiber involvement during the acupuncturist's manipulation for the de-qi response. Manual acupuncture activates all types of afferent fibers (A-beta, A-delta, and C) by inserting a needle into an acupoint, followed by twisting the needle by hand. The stimulation of MA comes from specific finger maneuvering that drives the needle's translation, rotation, or tremor. Hence, different MA manipulations can produce other therapeutic effects. Furthermore, even with the same manipulation, organisms' physiological changes or physicochemical reactions are diverse because of varying stimulation parameters such as frequency and depth [29]. To improve standards for reporting interventions in clinical trials of acupuncture, the STRICTA guidelines were designed.

Three trials used EA in the treatment group; a needle is inserted with stimulating current and is delivered to acupoints to excite A beta which induces an analgesic effect [27]. While another two studies used auricular acupuncture (AA) as a treatment intervention. Ear acupuncture or AA is a method in which thin needles are inserted at specific points on the outer ear to control pain and other symptoms. This results in activating the reticular formation and sympathetic and parasympathetic nervous systems to alleviate the pain and illness [30]. 

Acupoints pathophysiological dynamics is one of the most important concepts of acupuncture. They are located on any part of the body where there is a sensory nerve. Therefore, they appear anywhere since sensory nerves are distributed all over the body. The various effects depend on their connection to the effector organ through the brain. The effectiveness of acupoints may depend on the convergent inputs of somatic areas and the integrative function of the brain's neurons. The acupoints stimulation transmits signals along the appropriate nerves to the central nervous system (CNS). Then through the pain sensation conduction pathway where it can achieve analgesia. The integrative processes at different levels in the CNS between afferent impulses from pain regions and impulses from acupoints are essentially a manifestation of acupuncture analgesia [27,31].

Effectiveness of Acupuncture Versus Pharmacological Medication

Four trials with 451 participants compared acupuncture with pharmacologic medicine, including botulinum toxin A, topiramate, valproic acid, amitriptyline, beta-blockers, flunarizine, flunarizine + riboflavin, topiramate, pizotifen, duloxetine + coenzyme Q10, riboflavin, and a combination of another nutraceutical drug [2,15,21,22]. Two trials had participants with chronic migraines, and the other two had MWOA. Two studies showed a considerable mean reduction from baseline in the number of headache days in both acupuncture and topiramate treatment groups [15,22]. However, one study evaluated that acupuncture was statistically significantly more effective than topiramate in reducing the mean monthly number of moderate/severe headache days (-10.5 + 2.8 acupuncture vs. -7.8 + 3.6 topiramate; p< 0.1) [15]. Naderinabi et al.'s study showed that acupuncture reached a significantly more significant reduction VAS score compared with botulinum toxin and Valproate [2]. In addition, the acupuncture-treated group demonstrated a lower pain intensity and lower intake of rescue medication at six-months follow-up with no adverse events compared to those treated with valproic acid [21]. A recent study by Giannini et al. compared acupuncture with more appropriate pharmacological treatment for migraine prevention and revealed that headache frequency decreased significantly after treatment without differences between the two groups (time-effect: p < 0.001; group effect: p = 0.332; interaction time-group effects: p = 0.556) and the improvement persist after six months post-treatment [22].

Effectiveness of Acupuncture Versus Sham Acupuncture and Placebo AA

Comparison with sham acupuncture and placebo was made in 10 trials with 1,519 participants. Five studies used MA as a treatment intervention [9,11,19,23,24], two trials used AA [1,20], and the other three studies used EA [16-18]. Six out of 10 trials studied patients with MWOA, whereas the two studies had an acute attack, and three investigated either MWA or MWOA. The primary outcome in eight trials was evaluated by VAS [1,9,11,16,17,19,20,24] and the other two used number/frequency of HA attack [18,23]. 

Seven out of 10 trials observed a more significant reduction in the frequency of migraine attacks and headache intensity [9,11,17,18,23,24]. A study by Li et al. followed a significant improvement in VAS score after acupuncture treatment (P < 0.05), while SA showed insignificant improvement in VAS score and headache frequency (P > 0.05) [19]. Wang et al. study stated significantly fewer migraine days (RA: 5.2 ± 5.0; SA: 10.1 ± 7.1; P = 0.008), less severe migraine (RA: 2.18 ± 1.05; SA: 2.93 ± 0.61; P = 0.004), and increased pressure pain thresholds when MA group compared with the SA group [24]. Two studies showed that both the treatment and control group helped treat migraine; they remarkably alleviated the clinical symptoms of migraine (intensity of pain, attack frequency, and days with migraine) and improved the quality of life [9,17]. Li et al. reported that patients in the acupuncture groups had fewer days with a migraine; however, the differences between treatments were not significant (p > 0.05) [16].

Effectiveness of Acupuncture Versus Waiting-list or No Treatment

Two clinical trials investigated acupuncture in comparison to the waiting-list group with 192 participants [19,26]. Patients for one study had either MWA or MWOA, while the other only investigated patients with MWOA. The primary outcomes were the difference in the number of migraine days, VAS, and MIDAS. Both studies reported that MA showed significant therapeutic effects in VAS score and headache frequency improvement compared to the waiting-list group. The research by Musil et al. revealed that after 12 weeks of acupuncture, the number of migraine days was reduced by five-and-half days in the acupuncture and the two days in waiting-list control groups, a statistically significant inter-group difference of two migraine days (95% CI: −4 to −1). Likewise, a significantly greater percentage of responders to treatment was noted in the acupuncture versus no treatment groups at the six-month follow-up (81% vs. 36%; p>0.001) [25].

Safety

None of the included trials reported serious adverse effects.

Five out of 10 trials did not report any adverse effect [1,19,20,22,23]. Acupuncture treatment adverse effect was reported to be mild to moderate and primarily associated with local needle insertion (hemorrhage, subcutaneous hematoma, ecchymosis, leg weakness, hematoma, pain, and local paresthesia). One patient fainted during acupuncture treatment; her symptoms of dizziness and sweating disappeared after 15 minutes of rest [17].

Two studies reported AEs associated with valproate intake (nausea, constipation, abdominal pain, drowsiness, weight gain, itching, asthenia, anorexia, weight gain, tremor, insomnia, somnolence, and alopecia [2,21]. One study that used botulinum toxin as a control group had ptosis, facial masking, or asymmetry [2]. 

Summary of Findings

We conducted comparisons separately according to the characteristic of interventions and controls. Several trials showed that acupuncture was significantly more slightly effective than placebo and sham acupuncture. Real acupuncture demonstrated persistent, more remarkable, and clinically relevant benefits for migraine, reducing the pain intensity (VAS), the number of days with migraine, and migraine frequency. Acupuncture is just as effective and has fewer side effects than any western medicine with fewer side effects, which was discovered in four clinical trials [2,15,21,22]. Two trials showed that acupuncture has more therapeutic effects compared to no treatment [18,19]. 

Trials included in this review commonly stated that acupuncture has a good safety profile. Several studies reported adverse effects were mild to moderate and mainly related to local needle insertion.

Limitations 

There are several limitations in this review. We restricted our reviews to RCT published within 10 years and exclusively in the English language; hence, we cannot exclude the possibility of missing out on essential studies published before 2011 and other languages, especially those of Chinese literature. In addition, several of the included trials were assessed as high or unclear risk on several domains. A blinding acupuncturist is often challenging due to the unique nature of acupuncture intervention, but several studies were still judged as low risk. In addition, there was considerable heterogeneity among the studies: different techniques of acupuncture for interventions, multiple parameters for pain scale as outcomes measurement, and differences in the controls. These variations could influence the results of the trials. 

Implications of This Review Research

Future clinical trials should adhere to detailed standards to reduce the risk of bias to deliver evidence-based data about the efficacy and safety of acupuncture for migraine treatment. In addition, those trials should follow the STRICTA guidelines to clear the specific method of each intervention.

## Conclusions

There is an increasing number of systematic reviews and clinical trials about acupuncture for migraine treatment. Some showed potential advantages in reducing pain and improving the quality of life or less use of rescue medication with mild adverse effects than standard therapy. Despite limitations secondary to the low quality and methodological restrictions of included studies, acupuncture appears to be an emerging migraine treatment and prevention at par with medication. In addition, it has longer-lasting effects, is safe, seems to be cost-effective, and reduces drug intake with the possibility of severe unwanted adverse effects. Acupuncture can be recommended as an alternative or adjunct to drug treatment for patients suffering migraines. However, further clinical trials that utilized the STRICTA recommendation are still needed to strongly present an evidence-based strategy.
